# Clinical validation study of dried blood spot for determining everolimus concentration in patients with cancer

**DOI:** 10.1007/s00228-017-2394-0

**Published:** 2017-12-08

**Authors:** A. E. C. A. B. Willemsen, L. M. Knapen, Y. M. de Beer, R. J. M. Brüggemann, S. Croes, C. M. L. van Herpen, N. P. van Erp

**Affiliations:** 1 Department of Medical Oncology, Radboud university medical center, Geert Grooteplein Zuid 10, 6525 GA Nijmegen, the Netherlands; 2 Department of Clinical Pharmacy & Toxicology, Maastricht University Medical Center+, Maastricht, the Netherlands; 3 CAPHRI-Care and Public Health Research Institute, Maastricht University, Maastricht, the Netherlands; 4 Department of Pharmacy, Radboud university medical center, Nijmegen, the Netherlands

**Keywords:** Cancer, Everolimus, Dried blood spot, Pharmacokinetics, Therapeutic drug monitoring, Method agreement

## Abstract

**Purpose:**

Everolimus treatment is seriously hampered by its toxicity profile. As a relationship between everolimus exposure and effectiveness and toxicity has been established, early and ongoing concentration measurement can be key to individualize the dose and optimize treatment outcomes. Dried blood spot (DBS) facilitates sampling at a patients’ home and thereby eases dose individualization. The aim of this study is to determine the agreement and predictive performance of DBS compared to whole blood (WB) to measure everolimus concentrations in cancer patients.

**Methods:**

Paired DBS and WB samples were collected in 22 cancer patients treated with everolimus and analyzed using UPLC-MS/MS. Bland-Altman and Passing-Bablok analysis were used to determine method agreement. Limits of clinical relevance were set at a difference of ± 25%, as this would lead to a different dosing advice. Using DBS concentration and Passing-Bablok regression analysis, WB concentrations were predicted.

**Results:**

Samples of 20 patients were suitable for analysis. Bland-Altman analysis showed a mean ratio of everolimus WB to DBS concentrations of 0.90, with 95% of data points within limits of clinical relevance. Passing-Bablok regression of DBS compared to WB revealed no constant bias (intercept 0.02; 95% CI 0.93–1.35) and a small proportional bias (slope 0.89; 95% CI 0.76–0.99). Predicted concentrations showed low bias and imprecision and 90% of samples had an absolute percentage prediction error of < 20%.

**Conclusions:**

DBS is a valid method to determine everolimus concentrations in cancer patients. This can especially be of value for early recognition of over- or underexposure to enable dose adaptations.

**Electronic supplementary material:**

The online version of this article (10.1007/s00228-017-2394-0) contains supplementary material, which is available to authorized users.

## Introduction

The introduction of everolimus has brought significant benefit for patients with metastatic renal cell carcinoma (mRCC), metastatic HR+/HER2-breast cancer (mBC), and advanced or unresectable neuroendocrine tumors of pancreatic, gastrointestinal, or lung origin [1–4]. Currently, the treatment with everolimus in patients with cancer is not individualized and no therapeutic drug monitoring (TDM) is routinely being performed. The standard initial dose is 10 mg orally once daily, which may be reduced in case of toxicity or fragility [5]. This practice is in contrast to solid organ transplantation medicine, where doses of 0.75 to 1.0 mg twice daily are used and where TDM of everolimus, including dried blood spot (DBS) monitoring, to guide dosing has been incorporated in the standard care for over 10 years [6, 7]. Also, everolimus treatment of subependymal giant cell astrocytoma with tuberous sclerosis complex is individualized based on TDM [8].

Several arguments point to the use of TDM for everolimus in patients with cancer as well. A relationship between everolimus drug exposure and effectiveness and safety has been established in several studies [9–12]. Everolimus trough concentrations above 11.9 μg/L and below 26.3 μg/L, respectively, were associated with a threefold increase in progression-free survival and a fourfold decreased risk of toxicity in patients with breast cancer, kidney cancer, and neuroendocrine cancer [10]. Larger studies are required to further define the optimal therapeutic window. Everolimus has an interpatient pharmacokinetic variability up to 36–45% and shows dose proportional pharmacokinetics over the range of 5 to 10 mg once daily [5, 9, 13–15]. Given the exposure-effectiveness relationship, the narrow therapeutic index, the large interpatient variability, and linear pharmacokinetics, it seems important to guide everolimus dosing pharmacokinetically.

If TDM of everolimus is performed in patients with cancer, sampling with DBS can bring many advantages over venous sampling, as it is minimally invasive, simple, and flexible. After adequate training and with clear instructions, patients can perform DBS at home and sent their sample by regular mail to the laboratory for analysis. Also, physicians may benefit from the ease of the DBS sampling method, as it can provide them with analysis results before patients visit the outpatient clinic for their (routine) check-up [7]. As such, DBS is a promising alternative to venous sampling and it already has become increasingly common in other anticancer drugs [16–19]. The use of DBS to predict venous whole-blood (WB) everolimus concentration has been established for organ transplantation patients, in which the administered dosage of everolimus is much lower than in cancer patients [6, 7, 20, 21]. In patients with cancer, the agreement between everolimus DBS concentrations and WB concentrations is yet unknown. In order to enhance the implementation of DBS in clinical practice, this is the first clinical validation study, in which we aim to determine the agreement and the predictive performance of DBS compared to WB to measure everolimus concentrations in patients with cancer.

## Methods

### Study population

The current study is an observational pharmacokinetic study in patients aged > 18 years, treated with everolimus for any type of solid tumor at the Radboud university medical center (Radboudumc), an academic hospital in the Netherlands. No exclusion criteria were set since the study population intends to reflect a “real-life” group of patients with cancer treated with everolimus. As such, the dose and duration of everolimus treatment was not restricted for inclusion.

### Sampling and everolimus concentration

Each patient was sampled in the outpatient clinic during their routine follow-up, at one moment, while being on steady state (i.e., treated for at least 7 days). Patients were asked not to take everolimus at the day of the visit at home, but only directly after obtaining the WB and DBS samples. Two drops of capillary blood were sampled on the sampling paper in order to create the DBS samples in duplicate. To establish whether the difference in blood source (capillary vs. whole blood) is a cause of variation, an additional DBS was made from a drop of whole blood (DBS_wb_).

Since hematocrit might affect the quantification of everolimus in DBS due to inhomogeneity of the droplet on the paper, the hematocrit value of the WB samples was determined at the day the venipuncture and DBS took place. All samples per patient were collected within 10 min of each other by a dedicated physician.

Time after drug administration (interval between last dose intake and sampling) and dosing scheme were documented to estimate everolimus trough concentration (C_trough_), as described by Wang et al. [22].

### Bioanalysis

The DBS and DBS_wb_ samples were visually inspected and scored whether the spot size was adequately shaped and sized for analysis. If both spots were of correct size, the average concentration of the two samples was used. If both spots were of incorrect size, these samples were not used for further analysis. After scoring of spot size, a 7.5-mm disk from the central part of the blood spot was punched out from the sample paper. Bioanalysis of the WB, DBS, and DBS_wb_ samples was performed using two validated ultra performance liquid chromatography-tandem mass spectrometry (UPLC-MS/MS) methods. One method was used for the analysis of WB samples and another method was used to analyze the DBS and DBS_wb_ samples [23].

### Statistical analysis

The Clinical and Laboratory Standards Institute advises to study 40 samples for agreement analysis [24]. However, as everolimus is measured in WB and therefore in the same matrix as DBS and as no effect of hematocrit is expected, the expected variation in DBS measurements is smaller than in other DBS studies. Moreover, in transplantation patients, good agreement of everolimus measurement between DBS and whole blood has been shown previously [21]. Therefore, we performed a power analysis for Bland-Altman analysis to determine the sample size [25]. Assuming an expected mean of difference of 9%, with a standard deviation of 5% and a maximum allowed difference of 25%, and *α* of 0.05 and power of 0.80, we required samples of 20 patients. Two extra patients were recruited for the risk of invalid samples.

To study the level of agreement between everolimus concentrations in DBS, DBS_wb_, and WB, Bland-Altman analysis was performed [26]. In this analysis, we set limits of clinical relevance on a 25% range around the ratio of the two measurements. This range was chosen, as everolimus can be dose-adjusted in steps of 25% of the total dose. Passing-Bablok regression analysis was performed to detect constant and proportional bias, by analyzing the intercept and the slope of the regression line, respectively [27].

Furthermore, the DBS everolimus concentration was used to predict the measured WB concentration. With Passing-Bablok regression, the intercept and slope were determined using the whole population while excluding the data of the individual patient from whom the WB everolimus concentration is to be predicted. Subsequently, the intercept and slope were used to predict the WB everolimus concentration, based on the DBS concentration. This process was repeated for each individual patient. For analyzing the predictive performance, the following equations from the guideline of Sheiner and Beal [28] were used.

For bias:

1 $$ \mathrm{median}\  \mathrm{prediction}\  \mathrm{error}=\mathrm{median}\ \left(\mathrm{WBpred}\hbox{-} \mathrm{WB}\right) $$

2 $$ \mathrm{median}\  \mathrm{percentage}\  \mathrm{prediction}\  \mathrm{error}\ \left(\mathrm{MPPE}\right)=\mathrm{median}\left[100\%\times \left(\mathrm{WBpred}\hbox{-} \mathrm{WB}\right)\div \mathrm{WB}\right] $$

For imprecision:

3 $$ \mathrm{root}\  \mathrm{median}\  \mathrm{squared}\  \mathrm{prediction}\  \mathrm{error}\ \left(\mathrm{RMSE}\right)=\sqrt{\mathrm{median}}{\left(\mathrm{WBpred}\hbox{-} \mathrm{WB}\right)}^2 $$

4 $$ \mathrm{median}\  \mathrm{absolute}\  \mathrm{percentage}\  \mathrm{prediction}\  \mathrm{error}\ \left(\mathrm{MAPE}\right)=\mathrm{median}\left[100\%\times \left|\left(\mathrm{WBpred}\hbox{-} \mathrm{WB}\right)\left|\div \mathrm{WB}\right.\right.\right] $$

Values of MPPE and MAPE of < 15% were considered acceptable. Overall predictive performance was measured by the percentage of samples with an absolute percentage prediction error of < 20%. We set the criterion that at least 67% of samples should have a prediction error of < 20%, analogous to the criteria for cross validation of the European Medicines Agency (EMA) guideline on bioanalytical method validation [29].

All calculations were performed using Microsoft Office Excel (Microsoft Inc., Redmond, WA) and add-in Analyse-it statistics software, version 4.10.2 (Analyse-it Software, Ltd., Leeds, UK).

## Results

### Patients and everolimus concentrations

As planned, 22 patients were included, from June to December 2015. WB, DBS, and DBS_wb_ samples of 20 patients were included in the final analysis, since one set of samples were lost and the duplicate DBS samples of one patient was insufficient for analysis. The population consisted of 11 patients with mBC and 9 patients with mRCC. The median age at index date was 62.0 years (range 38–73) and the median everolimus dose was 10 mg (range 5–17.5 mg). The median hematocrit value of the WB samples was 0.35 L/L (range 0.25–0.45) (Table 1). The everolimus concentrations of the 20 analyzed samples ranged from 3.7 to 33.3 μg/L in DBS, from 3.3 to 31.2 μg/L in DBS_wb_ and from 3.6 to 28.5 μg/L in WB. When the C_trough_ concentrations were calculated for the individual patients using the equation of Wang et al., 55% of patients had a C_trough_ concentration below 11.9 μg/L and 15% a C_trough_ concentration above 26.3 μg/L [22].

**Table 1:** Baseline characteristics of patients

Baseline characteristics	*n* = 20
Age (years)	62 (38–73)
Sex (number (%))	
Male	8 (40%)
Female	12 (60%)
Weight (kg)	72.4 (48.5–90.2)
Hematocrit (L/L)	0.35 (0.25–0.45)
Everolimus daily dose (mg)	10.0 (5.0–17.5)
Tumor type (number (%))	
Breast cancer	11 (55%)
Renal cell carcinoma	9 (45%)

Data are median (range), unless stated otherwise

### Agreement between DBS, DBS_wb_, and WB everolimus concentrations

**Fig. 1:**
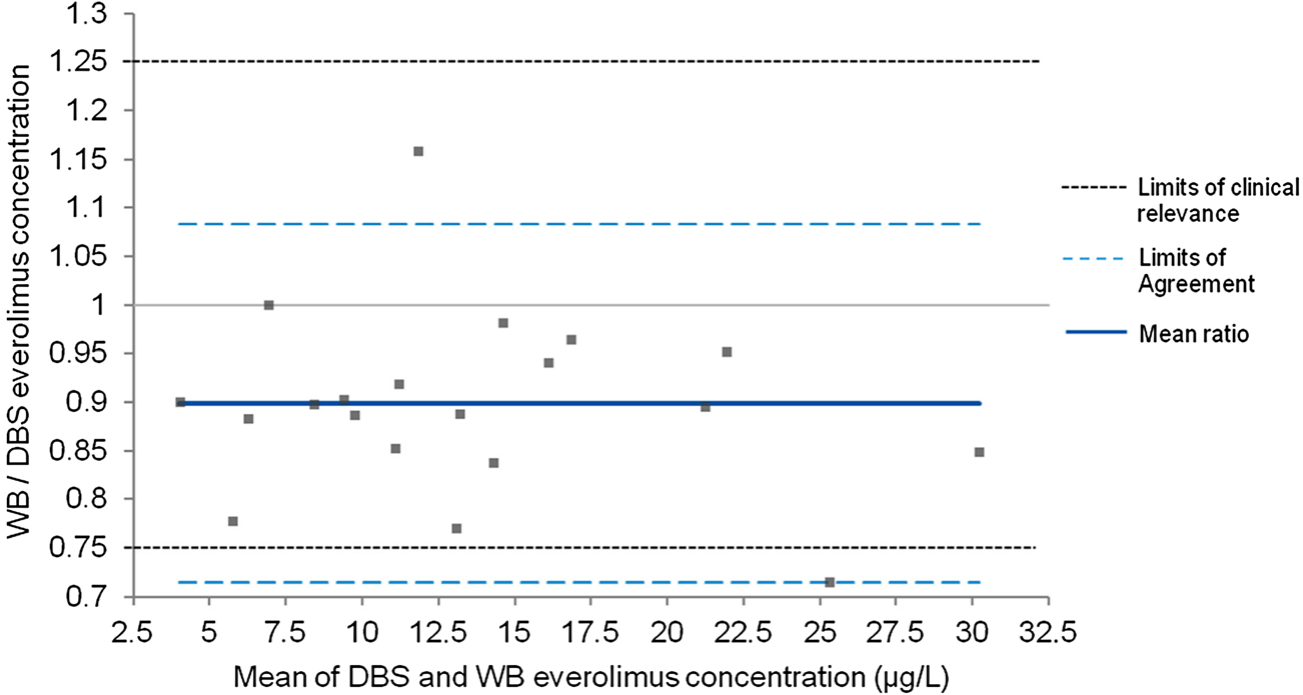
Bland-Altman plot of ratio between WB and DBS everolimus concentrations versus mean everolimus concentration

**Fig. 2:**
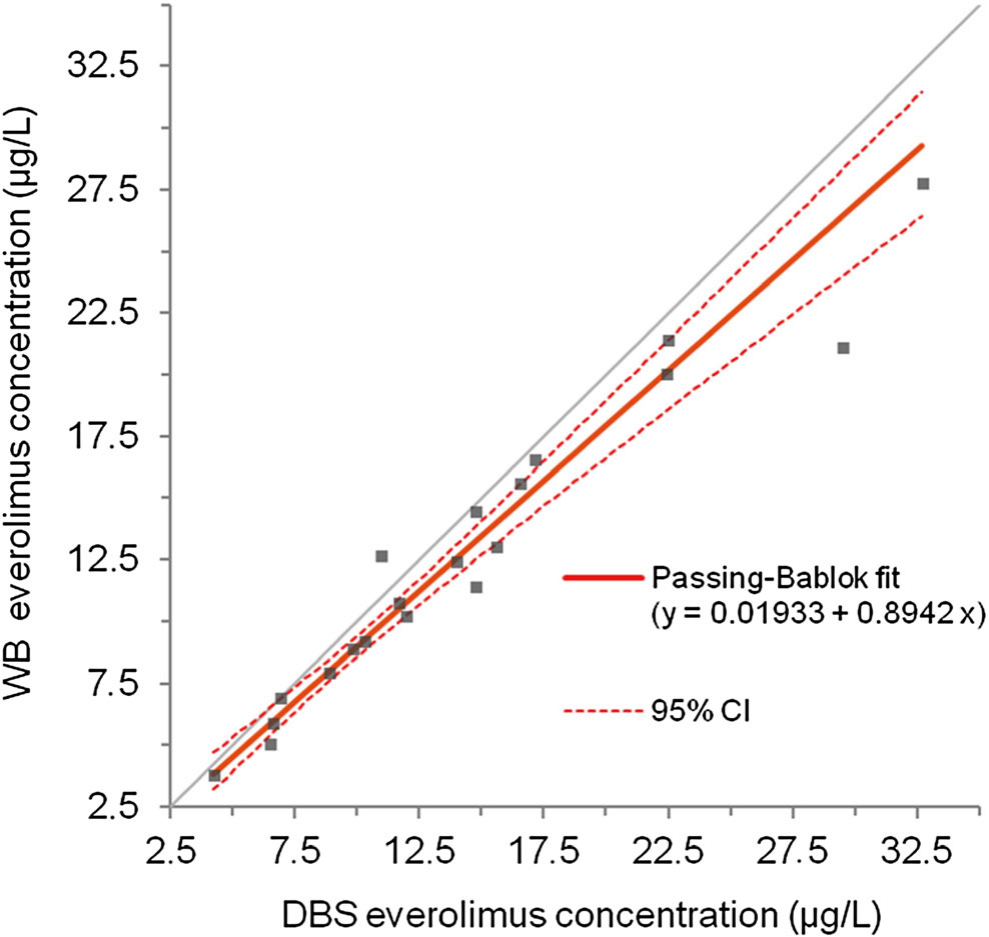
Passing-Bablok plot of everolimus concentrations from DBS and WB

Bland-Altman analysis was used to determine the level of agreement between DBS, DBS_wb_, and WB everolimus concentrations. The Bland-Altman plot with 95% limits of agreement (LoA) showed a small and balanced spread of relative differences between DBS and WB everolimus concentrations, with a mean ratio of everolimus WB to DBS concentrations of 0.90 (95% LoA 0.71–1.08). Only 1/20 (5%) of values fell outside the limits of agreement and outside the limits of clinical relevance (Fig. 1). Using Passing-Bablok regression analysis, a strong linear relationship was found between both methods, with a correlation coefficient of *r* = 0.97, thus explaining 95% of the variance (*r* ^2^ = 0.95). No significant constant bias was found, with an intercept close to zero (intercept estimate 0.02 μg/L; 95% CI − 0.93–1.35), and only a small proportional bias (slope estimate 0.89; 95% CI 0.76–0.99) (Fig. 2).

Agreement between DBS_wb_ and WB everolimus concentrations was comparable to the results of the DBS and WB analysis. Bland-Altman analysis showed a mean ratio of everolimus DBS_wb_ concentrations to WB concentrations of 0.92 (95% LoA 0.79–1.05). Passing-Bablok regression showed a coefficient of determination *r* ^2^ = 0.98, no constant bias (intercept − 0.17 μg/L; 95% CI − 1.37–0.51), and no proportional bias (slope estimate 0.93; 95% CI 0.87–1.04).

Everolimus concentrations of DBS and DBS_wb_ were similar, as the Bland-Altman plot showed a mean ratio of nearly 1 (mean ratio 0.98; 95% LoA 0.82–1.13). The Passing-Bablok regression of the two DBS sampling methods showed a coefficient of determination *r* ^2^ = 0.97, no constant bias (intercept 0.46 μg/L; 95% CI − 0.71–2.40) and no proportional bias (slope estimate, 0.93; 95% CI 0.80–1.03).

### Predictive performance of predicted everolimus WB concentrations compared with measured WB

**Fig. 3:**
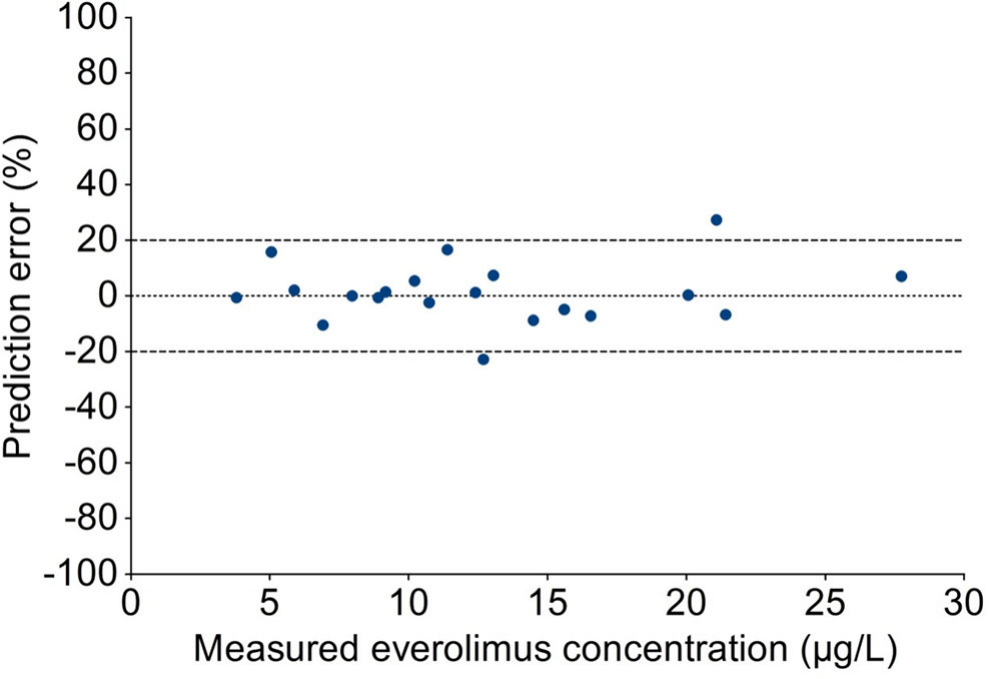
Percentage prediction error of predicted to measured everolimus concentration

Based on the DBS concentrations and using the intercept and slope, everolimus WB concentrations were predicted. The total error of this prediction is determined by bias (average difference between estimator and true value) and imprecision (variance of the estimator). Bias between the predicted and the measured everolimus concentration was negligible, with median absolute difference as measured by MPE of only 0.015 μg/L, and median relative difference as measured by MPPE of 0.035%. The imprecision of the predicted concentration was small, with RSME of 0.76 μg/L and median absolute percentage prediction error (MAPE) of 6.1%. These values are well within the acceptable limits of 15%. Overall predictive performance was good, as 90% of samples had an absolute percentage prediction error of < 20% (Fig. 3), which fell within the criterion of at least 67% of samples [29].

## Discussion

In this study, we have shown that the agreement of DBS with WB concentration measurements of everolimus in patients with cancer is very high over the entire concentration range. With DBS, results of 95% of samples fell within the limits of clinical significance, which would thereby lead to the same dosing advice as when WB measurement was used. The predictive performance of DBS was excellent, with negligible bias and small imprecision and good overall predictive performance, satisfying the EMA criteria for cross validation [29, 30]. Consequently, in view of the high agreement and excellent predictive performance, DBS is a valid method as a practical alternative for venous sampling to measure everolimus concentrations in patients with cancer. As such, these results confirm the results of a DBS validation study of everolimus in 55 transplant patients, where a similar slope and a somewhat larger intercept was found [21].

DBS_wb_ was determined in order to discriminate influences of the blood drop collection methods (capillary vs. whole blood) and material (filter paper vs. EDTA tube). As DBS and DBS_wb_ everolimus concentrations were nearly similar, it can be concluded that collection of blood by finger prick does not affect concentration measurements of everolimus.

Albeit not significantly, DBS and DBS_wb_ did show consistently higher everolimus concentrations compared to measuring everolimus in WB. Everolimus is strongly bound to erythrocytes (approximately 85% at the blood concentration range of 5–100 μg/L) [31, 32], and we speculate that this fraction can have a higher concentration at the center of the punch of the sampling paper. When hematocrit levels are low (≤ 0.20 L/L), chromatographic effects can play a role, resulting in inaccurately lower everolimus concentration measurements [33]. Especially DBS samples with high everolimus concentrations (> 20 μg/L) in combination with low hematocrit can provide inaccurate lower results [21, 34]. As hematocrit levels were ≥ 0.25 L/L for all patients, the impact of hematocrit on everolimus concentration measurement was not an issue in the population we investigated. However, awareness should be in place for everolimus measurements in patients with a hematocrit below 0.20 L/L. The collection of blood spots by volumetric absorptive microsampling is an alternative for DBS sampling that might overcome this issue, as it enables the collection of an accurate blood volume, independently of hematocrit levels [35].

It is important to note certain limitations of this study. First, the finger prick sampling was performed by a dedicated physician at the hospital, and since this situation does not reflect the at-home oncology sampling setting, the results cannot be extrapolated without further validation. Therefore, we recommend future studies to perform a validation of clinical utility with DBS cards sampled by patients themselves. However, we expect at-home sampling to be feasible when clear instructions and adequate training are provided, since previous literature has shown that 86 to 98% of the DBS samples obtained from patients were suitable for analysis [36–38]. Second, the number of analyzed DBS was relatively small. A smaller sample size leads to a wider confidence interval, resulting in a decreased power to detect a difference between two methods. However, this sample size is comparable to the sample size in other studies with DBS and oral anticancer agents [16, 39], while the agreement between DBS with WB for everolimus is markedly better than for other anticancer drugs, such as pazopanib and nilotinib [16, 39]. DBS shows a relatively high performance to measure everolimus concentrations, when compared to the performance of DBS for measuring other drugs. This can partially be explained as everolimus is measured in WB instead of plasma, and therefore has the same matrix as DBS. Moreover, good agreement of DBS and whole blood has been shown for everolimus in transplantation patients [21]. Therefore, we considered it appropriate to base our sample size on a power calculation using previously obtained data in transplantation patients treated with everolimus for our assumptions. This deems to be a legitimate approach, since our data indeed show highly similar results to the previous independently performed study. Third, only trough levels were analyzed. This did not lead to restrictions, however, as a wide range of everolimus concentrations were obtained.

In summary, it can be concluded that this is the first study that demonstrates the clinical validity of DBS sampling for analysis of everolimus concentration in patients with cancer. Measuring everolimus concentrations in patients with cancer is particularly important to identify patients with very low or high everolimus concentrations. In our study 70% of our patients had extrapolated everolimus trough concentrations that fell outside the proposed therapeutic window. In these patients, it is likely that dose adjustments can improve effectiveness or prevent toxicity. DBS is a patient-friendly and practical alternative for WB concentration measurements and in this study has shown to be accurate for the goal to individualize everolimus therapy in cancer patients. Implementation of standard everolimus DBS measurement early after start of treatment has the potential to improve clinical outcomes for patients with cancer treated with everolimus.

## Electronic supplementary material

ESM 1 (DOCX 17 kb)
