# Supplementary material for: Clinical validation study of dried blood spot for determining everolimus concentration in patients with cancer
Source: Eur J Clin Pharmacol. 2017 Dec 8;74(4):465–71. doi: 10.1007/s00228-017-2394-0 (PMC5849667; doi:10.1007/s00228-017-2394-0)
Supplement: Supplementary file 1 — (DOCX 17 kb) [file 228_2017_2394_MOESM1_ESM.docx]

## **Methods**

## Sampling

## The WB samples obtained from venipuncture were collected in 4 mL K_2_EDTA tubes, which then were stored at -40°C until analysis. For the DBS, a finger prick was performed using a 1.8 mm depth BD Microtainer Contact needle of 21g (Eysins, Switzerland) or the 2.0 mm depth BD Microtainer Contact lancet blade of 1.5 mm width (Eysins, Switzerland), in case of insufficient blood supply. For the DBS_wb_ 35 µL of WB from the K_2_EDTA WB sample was spotted in duplicate with a pipette onto the premarked circle on the sampling paper. The sampling paper used was Protein saver 903, ref no. 10531018, obtained from Whatman Gmbh (Dassel, Germany). The DBS and DBS_wb_ were dried for at least 15 minutes at room temperature, after which they were stored at room temperature in a closed envelope containing a 2 g silica gel sachet, until analysis.

*Sample preparation, extraction and bioanalysis*

## The sample preparation of the venous blood samples involves the precipitation of 200 µL EDTA blood with a 200 µL precipitation reagent (internal standard solution mixed with zinc sulphate heptahydrate 89mg/L in methanole in a 4:1 ratio). The supernatant is then transferred to the autosampler vial and 10 µL is injected into the ultra performance liquid chromatography-tandem mass spectrometry (UPLC-MS/MS). DBS sample preparation and extraction procedure were conducted according to methods that have been described previously [1].

For both UPLC-MS/MS methods, the sample was injected directly onto an analytical column (Waters Acquity UPLC BEH C18 1.7 µm, 2.1 mm x 50 mm), after which chromatographic separation was performed by gradient analysis using a Waters UPLC H-class system (Waters, Milford, Massachusetts, USA). The Xevo TQ-S micro Triple Quadrupole mass spectrometer fitted with an electrospray ionization source (Waters, Manchester, the UK) was used to analyze the samples. In both assays, the UPLC-MS/MS settings were grossly similar. The WB analytical run included a calibration curve of everolimus over the concentration range of 1-75 µg/L and quality control samples analyzed in duplicate at two different concentrations of 5.04 and 12.6 µg/L. The DBS analytical run included a calibration curve of everolimus over the concentration range of 3-75 µg/L and quality control samples analyzed in duplicate at three different concentrations of 6, 25 and 40 µg/L. Deuterium-labeled everolimus (everolimus-D4) was used as internal standard. For the WB and DBS assays, the within-day and between-day precisions were respectively ≤8.7% and ≤10.7%, and the within- and between day accuracies were respectively -6.2 to 11.7% and -4.4 to 7.6% [1]. For the DBS assay, the analytical results were sufficient for the hematocrit levels (0.25 – 0.50 L/L). The bioanalytical assays were validated according to the European Medicines Agency international guideline [2].

**References**

1 Knapen LM, Beer Yd, Brüggemann RJM, Stolk LM, Vries Fd, Tjan-Heijnen VCG, Erp NPv, Croes S (2017) Development and validation of an analytical method using UPLC–MS/MS to quantify everolimus in dried blood spots in the oncology setting. J Pharm Biomed Anal DOI <https://doi.org/10.1016/j.jpba.2017.10.039>

2 EMA. Guideline on bioanalytical method validation. 21 July 2011. <http://www.ema.europa.eu/docs/en_GB/document_library/Scientific_guideline/2011/08/WC500109686.pdf>. Accessed 2 Mar 2017.
